# Bempedoic Acid: An Emerging Therapy for Uncontrolled Low-Density Lipoprotein (LDL) Cholesterol

**DOI:** 10.3390/jcdd10050195

**Published:** 2023-04-27

**Authors:** Akshyaya Pradhan, Monika Bhandari, Pravesh Vishwakarma, Abhishek Singh, Marco Alfonso Perrone, Rishi Sethi

**Affiliations:** 1 Department of Cardiology, King George’s Medical University, Lucknow 226003, India; 2 Department of Cardiology and CardioLab, University of Rome Tor Vergata, 00133 Rome, Italy

**Keywords:** Bempedoic acid, LDL cholesterol, ATP citrate lyase, statin intolerance, uric acid

## Abstract

Atherosclerotic cardiovascular disease (ASCVD) is a silent epidemic, which is progressing relentlessly across the globe. Developing countries such as India have a high prevalence of dyslipidemia and consequently a huge burden of coronary artery disease (CAD) and ASCVD. Low-density lipoprotein is regarded as the primary culprit in the genesis of ASCVD, and statins are the first line therapy for LDL-C lowering. Statin therapy has unequivocally demonstrated the benefit of lowering LDL-C in patients across the spectrum of CAD and ASCVD. Muscle symptoms and worsening of glycemic homeostasis could be challenges with statin therapy, especially with the use of high doses. A large fraction of patients are also unable to achieve their LDL goals with statins alone in clinical practice. Moreover, LDL-C goals have become aggressive over years, necessitating a combination of lipid lowering therapies. PCSK-9 inhibitors and Inclisiran have emerged as robust and safe lipid-lowering agents, but parenteral administration and high cost precludes their widespread use. Bempedoic acid is a novel lipid-lowering agent working upstream of statins by inhibiting the enzyme ATP citrate lyase (ACL). The drug produces an average LDL lowering of 22–28% in statin-naïve patients and 17–18% when given to preexisting statin users. Because skeletal muscles lack the ACL enzyme, there is minimal risk of muscle-related symptoms. In combination with ezetimibe, the drug synergistically reduced LDL-C by 39%. Moreover, the drug has no adverse effect on glycemic parameters and lowers hsCRP (inflammation) like statin. The series of four randomized CLEAR trials, involving >4000 patients, have shown consistent LDL lowering across the spectrum of ASCVD patients with or without background therapy. The large and only cardiovascular outcome trial of the drug (CLEAR Outcomes) has recently demonstrated a 13% reduction of MACE at 40 months. Rise in levels of uric acid (four times) and acute gout (three times) are more common compared to placebo with the drug, owing to competitive renal transportation by OAT 2. In a nutshell, Bempedoic acid represents a value addition to the inventory of dyslipidemia management.

## 1. Introduction

India is in the midst of an atherosclerotic cardiovascular disease (ASCVD) epidemic that shows no signs of regression, probably due to the higher prevalence of dyslipidemia in the Indian population, with 79% of Indians having at least one lipid abnormality, including decreased high-density lipoprotein cholesterol (HDL-C) levels in 72.3% of subjects, hypertriglyceridemia in 29.5% of subjects, and elevated low-density lipoprotein cholesterol (LDL-C) levels in 11.8% of subjects [1]. A higher level of dyslipidemia is responsible for early incidence of coronary artery disease (CAD), about a decade earlier than in Western countries, especially in the young population; the same can be said for the 10–25% incidence of myocardial infarction (MI) reported before the age of 40 years in India. More than half of all CAD-related deaths occur before the age of 50 years [1]. 

In contrast, in Europe almost 80% of patients at high or very high risk of cardiovascular events do not reach the LDL-C levels recommended by European guidelines [2]. The cardiovascular risk of patients with hypercholesterolemia is significantly underestimated, and suboptimal use of lipid-lowering therapies persists, with the result that a substantial percentage of patients remain at high residual risk of cardiovascular events, as demonstrated by the SANTORINI study [3].

## 2. Role of LDL-C in Development of ASCVD

Increased LDL-C is the primary cause of atherosclerotic cardiovascular disease (ASCVD), and its retention within the arterial wall is the key initiating event in atherogenesis. Meta-analyses of over 200 prospective cohort studies, Mendelian randomization studies, and randomized controlled trials involving over 2 million participants with over 20 million person-years of follow-up and over 150,000 ASCVD events have revealed a remarkably consistent dose-dependent log-linear relationship between the absolute magnitude of vasculature exposure to LDL-C and the risk of ASCVD. According to the INTERHEART study, LDL-C is as important a risk factor in Indians, as it is in other ethnic groups [4].

## 3. Role of Statins for CVD Prevention

Consequently, optimal dyslipidemia management focusing on LDL-C target achievement, as well as control of other risk factors, is critical to arresting the ASCVD epidemic. In multivariable regression analyses, each 50% incremental reduction in LDL-C on top of statins was associated with a further 29% reduction in the risk of major adverse cardiovascular events (MACE) [5].

Cholesterol Treatment Trialists’ (CTT) meta-analysis of randomized trials showed that every level of LDL-C reduction can reduce MACE events. Even 1 mmol/L of LDL-C reduction can reduce the 5-year incidence of MACE events by about 20% with standard statin therapy, and with higher-intensity statin therapy, it reduces further MACE events proportionately with LDL-C reduction. Hence, even post standard statin therapy, if LDL-C remains uncontrolled, then it should be further reduced through lipid-lowering therapy (LLT) [6].

In the last few decades, statins have shown significant benefits in terms of MACE events reduction in patients with prior history of CAD or atherosclerotic cardiovascular disease (ASCVD) through several large randomized controlled trials (RCTs). A post hoc analysis of the study, Treating to New Targets (TNT), showed a significant reduction in the risk of MACE with descending achieved levels of on-treatment LDL-C. A proportionate reduction of all-cause mortality and CV death to the continuation of statin therapy and LDL-C reduction was observed [7].

## 4. LDL-C Goals Revision as per ESC 2019 (vs. ESC 2016)

The European Society of Cardiology (ESC) along with the European Atherosclerosis Society (EAS) in 2019 released updated recommendations for primary prevention with statin therapy, which had several changes as compared to the 2016 guidelines [2,8]. These changes were:

- An updated Systematic Coronary Risk Evaluation (SCORE) risk assessment chart was introduced, which was also applicable to individuals above age 65 years.
- Class I/A recommendations for statin therapy were expanded by lowering the untreated LDL-C levels eligible for treatment.
- Finally, Class I/A statin recommendation was also provided for individuals aged 66–75 years.

ESC/EAS 2019 guidelines the revised LDL-C goal for very-high-risk ASCVD patients to <55 mg/dL and patients with second CV events within 2 years of index CV event, whose LDL-C target should now be <40 mg/dL. LDL-C goals are revised based on clinical studies of PCSK9 inhibitors and ezetimibe. According to revised recommendations, for any statin-naïve ACS patients, high-intensity statin should be started as early as possible for optimal benefit in CV events reduction.

Another major difference between the 2019 and 2016 ESC/EAS guidelines is the new lower LDL-C criteria for initiating risk-based statin treatment. These have been lowered substantially in the 2019 guidelines to 2.6 mmol/L (100 mg/dL) instead of 4.0 mmol/L (155 mg/dL) for SCORE risk of 5–10% and 1.8 mmol/L (70 mg/dL) instead of 2.6 mmol/L (100 mg/dL) for SCORE risk of ≥10% (Table 1). Thus, the revised 2019 guidelines indirectly endorse the fact that statins are not only LDL-C lowering drugs but also risk-lowering drugs that are effective even in subjects with relatively low cholesterol levels [8].

Similarly to the ESC guideline recommendation, the Lipid Association of India (LAI) 2016 recommended LDL-C targets of <50 mg/dL and <70 mg/dL in very-high-risk and high-risk CVD patients, respectively [1]. Recently, in 2020, LAI added one more risk category, the extreme-risk group, with subcategories of A and B. In group A, a < 50 mg/dL LDL-c target is indispensable, while <30 mg/dL must be pursued after a detailed risk–benefit discussion between the physician and patient. Extreme risk group B characteristics are patients CAD† with 1 of the following: either diabetes + polyvascular disease/>3 major ASCVD risk factors/target organ damage, or recurrent ACS (within 12 months) despite being on LDL-C goal, or homozygous familial hypercholesterolemia. For such patients, the LDL-c target should be <30 mg/dL [9].

## 5. Poor LDL-C Goals Attainment Based on Revised Guidelines: DA VINCI and DYSIS II

Assessment of the usage of lipid-lowering therapy (LLT) and attainment of the 2016 LDL-C goals have focused on patients with coronary artery disease in secondary care settings, but overall goal attainment was low. The 2019 ESC/EAS guideline update recommends even lower LDL-C goals for the very high risk, high risk, and moderate risk categories. Based on these revised recommendations, it is still unclear whether these lower goals can be attained and whether optimizing statin alone is sufficient to achieve these lower goals [2,8].

**Figure 1:**
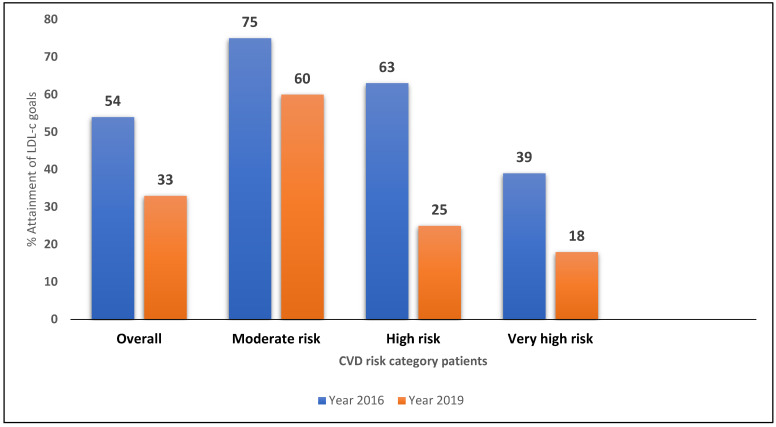
DA VINCI Study: % attainment of LDL-C goals based on CVD risk category (ESC 2016 vs. ESC 2019).

The EU-Wide Cross-Sectional Observational Study of Lipid-Modifying Therapy Use in Secondary and Primary Care (DA VINCI study) study revealed that amongst the 5888 enrolled patients, including primary and secondary prevention patients, only half achieved their risk-based 2016 LDL-C goals, while in the same patients only 33% achieved their risk-based 2019 goals. Even irrespective of CV risk category, there is significantly low attainment of 2019 LDL-C goals compared to 2016 goals (Figure 1) [10].

The Dyslipidemia International Study II (DYSIS II) of 320 acute coronary syndrome (ACS) patients showed very low attainment of LDL-C goals after usage of LLT. In this 4-month study, about 6 out of 10 ACS patients were treated with LLT prior to the acute event, and despite that, 26.7% for the initial LLT group and 24.1% for the no-LLT group achieved their LDL-C goals, though the dosage was still low and there was little use of combination therapy [11].

## 6. Residual Cardiovascular (CV) Risk Post Statin Therapy

The deposition of atherogenic lipoproteins is a major contributing factor for the development of atherosclerotic cardiovascular disease (ASCVD). For this condition, cumulative evidence from over two decades, involving more than 150,000 patients in clinical trials, has demonstrated the beneficial efficacy of statin in primary and secondary CV events reduction [12].

All major international and national guidelines recommend statin as first-line therapy for CVD, including stroke as well. However, despite the optimal use of statins, observations from various clinical trial data show higher residual CV risk in all patients treated with statins, despite optimal LDL-C reduction, which highlights the necessity of reevaluating other non LDL-c atherogenic parameters associated with triglyceride-rich lipoprotein (TGRLP), such as apolipoprotein B, very-low-density lipoprotein (VLDL), intermediate-density lipoprotein (IDL), lipoprotein(a) (Lp(a)), and so on [13].

## 7. Optimization of Uncontrolled LDL-C Management through Non-Statin Therapy

The DA VINCI study clearly demonstrated that amongst the patients receiving LLT, less than 50% of the high-/very-high-risk primary and secondary prevention patients achieved the 2016 LDL-C goals, with nearly 1/5th achieving the lower 2019 goals. Thus, despite optimized statin usage, a gap between guideline-recommended LDL-C goals and their actual clinical attainment under real-world clinical care exists. This mandates the utilization of non-statin LLT in combination with statins for the highest-risk patients [9]. The DA VINCI data provides further insights into the potential benefits that can be achieved by combination therapy. Nearly 21% of ASCVD patients receiving moderate- to high-intensity statin regimens with ezetimibe achieved LDL-C levels below 1.4 mmol/L. In addition, for those who received PCSK9 inhibitors in combination with other LLT, 58% attained LDL-C levels below 1.4 mmol/L. Thus, the addition of non-statin LLTs in combination with optimized statins is most likely needed to reduce these gaps for the patients at highest risk [8].

## 8. Bempedoic Acid: A Novel Non-Statin Lipid-Lowering Therapy

Apart from statins, cholesterol absorption inhibitors (ezetimibe), proprotein convertase subtilisin/kexin type 9 (PCSK9) inhibitors (evolocumab and alirocumab), and inclisiran (long-acting small interfering RNA (siRNA)) have emerged as non-statin LLTs. However, the cholesterol absorption inhibitors possess only modest LDL-C-lowering efficacy, in the range of 15–20%. Meanwhile, the PCSK-9 inhibitors and inclisiran, though they produce robust LDL-C reduction up to 50–60%, have a high cost burden and can be administered only subcutaneously. Bempedoic acid (BemA, ETC-1002 or 8-hydroxy-2,2,14,14-tetramethylpentadecanedioic acid) is a novel, non-statin, once daily, oral drug that has been developed for the treatment of primary hyperlipidemia in patients requiring additional lipid lowering [14].

Bempedoic acid is approved in the USA, EU, and India by the respective regulatory authorities. The US Food and Drug Administration (FDA) approved it as an adjunct to diet and maximally tolerated statin therapy for the treatment of adults with heterozygous familial hypercholesterolemia or established atherosclerotic cardiovascular disease who require additional lowering of LDL [15]. The Drugs Controller General of India (DCGI) has also approved bempedoic acid as per the USFDA-labelled indications. The Committee for Medicinal Products for Human Use (CHMP) of the European Medicines Agency (EMA) recommended approval of bempedoic acid in adults with primary hypercholesterolaemia (heterozygous familial and non-familial) or mixed dyslipidemia, as an adjunct to diet in combination with a statin or other lipid-lowering therapies in patients unable to reach LDL-C goals with the maximum tolerated dose of a statin, or alone or in combination with other lipid-lowering therapies in patients who are statin-intolerant or for whom a statin is contraindicated [16].

## 9. Mechanism of Action of Bempedoic Acid

**Figure 2:**
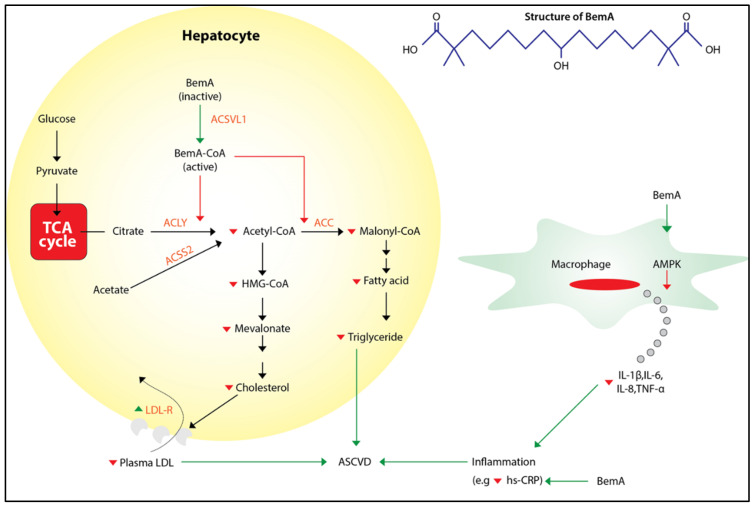
Mechanism of action of bempedoic acid [16]. (ACC: acetyl-CoA carboxylase, ACSS2: acetyl-CoA synthetase 2, AMPK: adenosine monophosphate-activated protein kinase, hs-CRP: high-sensitivity C-reactive protein, IL: interleukin, TNF-α: tumor necrosis factor-α; BemA: bempedoic acid, ACSVL1: very-long-chain acyl-CoA synthetase1, LDL-R: LDL-C receptor, ACLY: ATP-citrate lyase.)

Bempedoic acid is a prodrug that is converted into bempedoyl CoA, its active form, by the enzyme very-long-chain acyl-CoA synthetase-1 (ACSVL1). The ACSVL1 enzyme is expressed mainly in the liver and kidney, but not in the skeletal muscle or other tissues; this restricts the activity of bempedoic acid almost exclusively to the liver [17]. This drug inhibits de novo lipid synthesis in vitro and improves serum lipid profile in vivo via inhibition of acetyl-CoA carboxylase (ACC) and ATP-citrate lyase, which leads to the attenuation of fatty acid and sterol synthesis in the liver, respectively. Additionally, following bempedoic acid treatment, the lowering of hepatic cholesterol stimulates an upregulated expression of LDL-C receptor (LDL-R), which permits an increased uptake of plasma LDL-C. Clinical studies have also shown that bempedoic acid also inhibits inflammatory responses in macrophages in vitro and significantly reduces high-sensitivity C-reactive protein (hs-CRP). Conclusively, the lipid-lowering and anti-inflammatory activity brings about a reduction in the progression of atherosclerotic cardiovascular disease (ASCVD) (Figure 2) [18].

## 10. Pharmacokinetics of Bempedoic Acid

Bempedoic acid is well absorbed by the gastrointestinal tract without a food effect and achieves maximal plasma concentration within almost 3.5 h, with high plasma protein binding (99%). The drug has a long plasma half-life (approximately 21 h), allowing once-daily dosing. It is reversibly converted to active metabolite (ESP15228), followed by a glucuronide conjugation, and mainly excreted by the kidney (70%) as an inactive form. No dose reduction is necessary for patients with mild-to-moderate liver dysfunction, though data for patients with severe liver impairment is lacking. Dose adjustment is not needed despite the presence of an increased area under the concentration–time curve (AUC) in patients with mild-to-moderate renal impairment [18].

## 11. Bempedoic Acid—Efficacy Analysis from Clinical Trials

Four phase III clinical trials have been conducted to determine the effect of bempedoic acid (BA) in patients with dyslipidemia, known as the CLEAR (Cholesterol Lowering via ETC-1002, an ACL (ATP-citrate lyase)-Inhibiting Regimen) trials. The CLEAR studies can be classified into two main groups based on the studied population. The first group uses bempedoic acid add-ons to statins in patients who have failed to achieve LDL-C targets (i.e., CLEAR Harmony and CLEAR Wisdom) [19,20]. The second group uses bempedoic acid in patients with statin intolerance (i.e., CLEAR Serenity and CLEAR Tranquility) [21,22] (Table 2).

In a pooled analysis of 10 eligible trials, it was shown that bempedoic acid treatment resulted in greater lowering of the low-density lipoprotein cholesterol level than the placebo group (mean difference –23.16%, 95% CI –26.92% to –19.04%; *p*< 0.00001). In the pooled analysis, considerable heterogeneity among studies (I^2^ = 82%, *p* < 0.00001) was also observed. Significant reductions were also observed for non-HDL-C level (mean difference –18.30%, 95% CI –21.65% to –14.95%; *p* < 0.00001), TC level (mean difference –14.62%, 95% CI –17.08% to –12.16%; *p* < 0.00001), apoB level (mean difference –14.77%, 95% CI –16.85% to –12.70%; *p*< 0.00001), and HDL-C level (mean difference –3.80%, 95% CI –5.54% to –2.06%; *p* < 0.00001). In long-term evaluations over 52 weeks, bempedoic acid demonstrated consistent improvements in lipid parameters and biomarkers [23].

**Figure 3:**
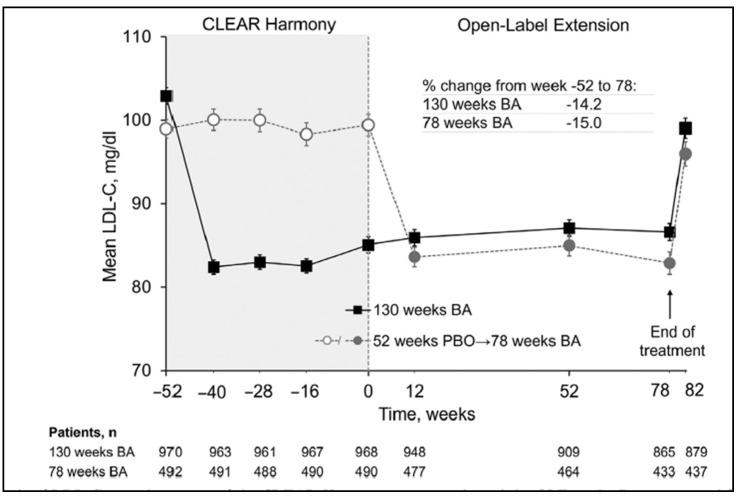
LDL-C levels observation over the course of the CLEAR Harmony parent study and the OLE study. [22] (BA: bempedoic acid, PBO: placebo).

In the CLEAR Harmony Open Label Extension (OLE) study that lasted 78 weeks, patients who received placebo in the parent study were switched over to bempedoic acid. At the end of the study, mirroring reductions were observed in patients who received bempedoic acid in the parent study who remained stable through 78 weeks of therapy, with comparable safety parameters. This showed that bempedoic acid was generally well tolerated and demonstrated sustained efficacy with up to 2.5 years of continuous treatment (Figure 3) [24].

**Figure 4:**
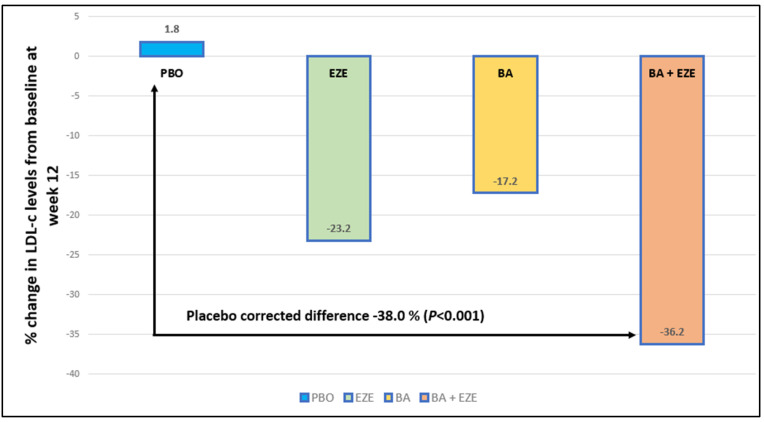
Change from baseline to week 12 in low-density lipoprotein (LDL) cholesterol in fixed-dose combination of bempedoic acid plus ezetimibe, ezetimibe alone, bempedoic acid alone, and placebo (25). (BA: bempedoic acid, EZE: ezetimibe, PBO: placebo, BA + EZE: bempedoic acid + ezetimibe.)

In a phase III study of a bempedoic acid (180 mg) and ezetimibe (10 mg) fixed-dose combination (FDC), 301 patients with hypercholesterolemia and a high risk of CVD receiving maximally tolerated statin therapy were randomized (2:2:2:1) to treatment with the FDC, bempedoic acid 180 mg, ezetimibe 10 mg, or placebo added to stable background statin therapy for 12 weeks. The FDC lowered low-density lipoprotein cholesterol (–36.2%) significantly more than placebo (1.8% (placebo-corrected difference –38.0%); *p* < 0.001), ezetimibe alone (–23.2%; *p* < 0.001), or bempedoic acid alone (–17.2%; *p* < 0.001) (Figure 4). Improvements with the fixed-dose combination were also observed in secondary efficacy endpoints, including high-sensitivity C-reactive protein (hs-CRP), with a similar safety profile compared to other groups [25].

Metanalysis of six RCTs with a total of 3956 patients and 52 weeks of follow-up from Lin Y et al. showed no difference in major adverse cardiovascular events (MACE) for bempedoic acid vs. placebo (OR 0.84; 95% CI 0.61 to 1.15), with similar events of all-cause mortality (OR 2.37; CI 0.80 to 6.99) and CV mortality (OR 1.66; CI 0.45 to 6.04). Beneficial trends for non-fatal MI (OR 0.57; CI 0.32 to 1.00) and lower risk of new-onset or worsening diabetes (OR 0.68; CI 0.49 to 0.94) was seen in the bempedoic acid group, along with higher risk of gout and worsening of renal function [26].

In patients with various factors including statin absence, female sex, diabetes history, ezetimibe use, and higher high-sensitivity C-reactive protein level, bempedoic acid achieved LDL-C reductions comparable to a moderate- or high-intensity statin. In a post hoc analysis of four phase III trials, BA’s lowering of LDL-C levels was comparable to a moderate- or high-intensity statin (≥30%) in 28.9% of patients at week 12; this result was observed in half of statin-naïve patients [27].

Currently, few guidelines have recommended the use of bempedoic acid in familial heterozygous hypercholesterolemia or clinical ASCVD patients who are already on the maximally tolerated dose of statin plus ezetimibe [28,29]. The Polish Lipid Association (PLA) guideline recommended the use of BA in statin-intolerant patients at any dose (even after rechallenge), either as a monotherapy or along with ezetimibe (class of recommendation IIb and level of evidence B) [30].

## 12. Effect of Bempedoic Acid on Atherogenic Lipids and Inflammation

Bempedoic acid has a unique mechanism of action of ATP citrate lyase (ACL) enzyme inhibition, and along with statin, it inhibits cholesterol synthesis, up-regulates LDL receptors, and reduces circulating LDLc levels. Various clinical studies have also shown that it inhibits inflammatory responses in macrophages in vitro and significantly reduces high-sensitivity C-reactive protein (hs-CRP), apolipoprotein B, and non-HDLc, which may contribute to the prevention of further ASCVD events in dyslipidemia patients [18].

Masoon W et al. demonstrated in a pooled metanalysis of seven trials of bempedoic acid (3892 patients) a significant percentage reduction in apolipoprotein B levels (−14.3% (CI 95% −16.4 to −12.1); *p* < 0.05), non-HDL-C levels (−15.5% (CI 95% −18.1 to −13.0); *p* < 0.05), and hs CRP (−23.4% (CI 95% −32.6 to −14.2); *p* < 0.05; I^2^ = 69%) with the use of bempedoic acid. The sensitivity analysis showed that the results were robust [31].

**Figure 5:**
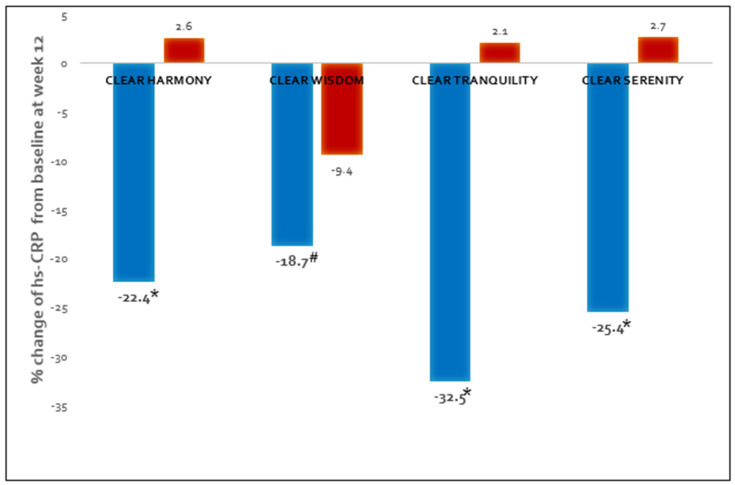
Change from baseline to week 12 in levels of high-sensitivity C-reactive protein (hs-CRP) with bempedoic acid treatment in CLEAR group phase III studies (* *p* <0.001; # *p* <0.04 [19,20,21,22]).

In a group of CLEAR phase III clinical trials, bempedoic acid showed significant improvement of hs-CRP at week 12 in HeFH with/without ASCVD or statin-intolerant patients (Figure 5) [19,20,21,22].

## 13. Bempedoic Acid Safety Analysis

In a pooled analysis of four phase III randomized trials, including 3621 patients, exposure-adjusted TEAE rates were similar for the bempedoic acid and placebo groups (87.1/100 and 82.9/100 person-years (PY), respectively). TEAEs leading to discontinuation rates were 13.4/100 and 8.9/100 PY for bempedoic acid vs. placebo, while myalgia was the most common event for discontinuation of therapy, which was lower but comparable in treatment group vs. placebo [32]. These observations also support the unique mode of action of bempedoic acid; as a prodrug, it is converted into its active form by very-long-chain acyl-CoA synthetase-1 (ACSVL1), an enzyme highly expressed in hepatocytes but not in skeletal muscle. For that reason, it is not expected to cause muscle-related adverse events, which are instead typically associated with the use of statins, as observed in pooled analysis of four phase III clinical trials, thus fostering the interest in this drug as a promising option for the treatment of statin-intolerant patients as well [33].

Bempedoic acid was associated with small mean increases in uric acid levels at week 12 (mean change 0.82 mg/dL vs. –0.02 mg/dL) vs. placebo, which was obvious in the first 4 weeks of treatment, was sustained during treatment, and was reversible after treatment discontinuation. The incidence rate of gout was 1.6/100 vs. 0.5/100 PY in the bempedoic acid vs. placebo groups. Patients with a prior medical history of gout had a higher incidence of gout during bempedoic acid treatment, compared to those with no history of gout (11.0% vs. 8.0%). Changes in uric acid levels were not influenced by baseline renal function. These laboratory abnormalities were more obvious during the first month of treatment, but they were stable and reversible later as well [32].

## 14. Effect of Bempedoic Acid on Glycemic Status

Bempedoic acid is associated with lower incidence of new onset of diabetes mellitus, observed in phase III clinical trials, as well as in pooled analysis of 3000 patents, with a lower annual rate of new onset of diabetes in normoglycemic and prediabetes patients in the bempedoic acid group compared to placebo (0.3% vs. 0.8 and 4.7% vs. 5.9%, respectively), with a significant reduction of HbA1c as well, over a median follow-up of 1 year [34].

## 15. Use of Bempedoic Acid along with Other Statins

Bempedoic acid is associated with fewer events of myalgia and related complications, but its usage with simvastatin >20 mg or pravastatin >40 mg dose should be avoided to reduce drug–drug-interaction-induced serious muscle-related events, such as tendon rupture, myopathies, and so on [35]. The pooled analysis of four CLEAR series trials discussed above has also shown a lower incidence of myalgia with bempedoic acid compared to placebo (1.5/100 person years vs. 2.0/100 person years) [32].

## 16. Effect of Bempedoic Acid on Uric Acid Level

In clinical trials, bempedoic acid was associated with elevations in serum uric acid (in 1.5% of patients), causing new-onset gout or precipitating gout flares in patients with pre-existing gout; for this reason, in patients with known hyperuricemia or gout, it has to be used with caution [35].

## 17. Bempedoic Acid in Patients with CKD and Hypertension

In a pooled analysis of four phase III studies with a total 3619 patients, including stage 1 (22%), stage 2 (63%), and stage 3a + b (15%) renal function at baseline, LDL-C lowering of BA was similar across renal function subgroups, with significant reductions of 20.8%, 18.8%, and 21.1% at week 12 from baseline in each subgroup, respectively, along with significant apoB reduction. The overall pattern of adverse events and creatinine levels was generally consistent within each renal function subgroup [36]. In a similar pooled analysis, 78% of patients had hypertension, with almost 19% LDL-C reduction at 12 weeks with BA treatment. That response was the same across patients with or without hypertension. BA was generally well tolerated with a consistent safety profile across treatment groups [37].

## 18. Bempedoic Acid—Long Term Cardiovascular Outcome Trial

Despite being a lipid-lowering drug, the effect of bempedoic acid on cardiovascular outcomes had not been evaluated until now. The Cholesterol Lowering via Bempedoic Acid, an ACL-Inhibiting Regimen (CLEAR) Outcomes trial, a phase III randomized clinical trial, has recently shown significant a reduction in four-point major adverse cardiovascular events (composite of death from cardiovascular causes, nonfatal myocardial infarction, nonfatal stroke, or coronary revascularization) by 13% after 40 months of therapy. The placebo-controlled trial enrolled 13,970 patients with statin intolerance who were at high risk of ASCVD and/or had elevated LDL-C level. Simultaneously, there were significant reductions in three-point MACE (defined as death from cardiovascular causes, nonfatal myocardial infarction, or nonfatal stroke), nonfatal MIs, and Coronary revascularizations by 15%, 23%, and 19%, respectively [38]. Unfortunately, the drug had no significant effect on the occurrence of nonfatal stroke and death from cardiovascular causes when analyzed separately. The incidences of gout and cholelithiasis were higher with bempedoic acid than with placebo, as were the incidences of small increases in serum creatinine, uric acid, and hepatic-enzyme levels. In a nutshell, this drug was found safe and significantly effective in terms of MACE reduction. Interestingly, the trial enrolled a high population of female participants (almost half). This is of pivotal importance for two reasons: first, females are generally underrepresented in clinical trials; and second, they generally are at high risk of statin intolerance.

## 19. Conclusions

Bempedoic acid is first in class for drugs with unique modes of action of ACL inhibition. In the current era of recent Indian and international guidelines with more stringent LDL-C goal criteria, there is a need for this type of molecule, with the potential of a further ~20% LDL-C reduction post maximally tolerated dose of statin treatment. In a pooled analysis of phase III clinical trials, it has been observed as a significantly effective and well-tolerated molecule, even in statin-intolerant patients. The recently published CLEAR Outcomes trial has demonstrated that bempedoic acid also reduces CV events in patients with statin intolerance, establishing the pivotal role of bempedoic acid in the emerging non-statin lipid-lowering therapy armamentarium.

## Figures and Tables

**Table 1:** Statin eligibility criteria (Class I/A recommendations) according to the 2016 and 2019 ESC/EAS dyslipidemia guidelines.

2016 ESC/EAS Guidelines	2019 ESC/EAS Guidelines
**Lipid-based:**	**Lipid-based:**
LDL-C > 6 mmol/L (232 mg/dL)	LDL-C > 4.9 mmol/L (190 mg/dL)
or	or
TC > 8 mmol/L (309 mg/dL)	TC > 8 mmol/L (309 mg/dL)
**Risk-based:**	**Risk-based:**
Age 40–65 years	Age 40–75 years
LDL-C ≥ 4.0 mmol/L (155 mg/dL)	LDL-C ≥ 2.6 mmol/L (100 mg/dL)
SCORE 5% to <10%	SCORE 5% to <10%
Or	Or
Age 40–65 years	Age 40–75 years
LDL-C ≥ 2.6 mmol/L (100 mg/dL)	LDL-C ≥ 1.8 mmol/L (70 mg/dL)
SCORE ≥10%	SCORE ≥10%
Or	Or
Diabetes	Diabetes
Or	Or
Non-dialysis dependent CKD and eGFR <60 mL/min/1.73 m^2^	Non-dialysis dependent CKD and eGFR <60 mL/min/1.73 m^2^

(CKD: chronic kidney disease, EAS: European Atherosclerosis Society, eGFR: estimated glomerular filtration rate, ESC: European Society of Cardiology, LDL-C: low-density lipoprotein cholesterol, TC: total cholesterol).

**Table 2:** Summary of major outcomes of CLEAR group of trials (phase III trials of bempedoic acid [19,20,21]).

Study Name	Population Studied	Intervention	LDL-C Reduction (Placebo Corrected Difference) at 12 Weeks
**Bempedoic acid add-on to statin**			
CLEAR Harmony	ASCVD and/or HeFH Taking maximally tolerated statin with or without other lipid-lowering agents ^#^ LDL-C ≥ 70 mg/dL	Bempedoic acid 180 mg (*n* = 1488) versus placebo (*n* = 742)	−18.1%
CLEAR Wisdom	ASCVD and/or HeFH Taking maximally tolerated lipid lowering therapy LDL-C ≥ 70 mg/dL	Bempedoic acid 180 mg (*n* = 522) versus placebo (*n* = 257)	−17.4%
**Bempedoic acid in statin-intolerant patients**			
CLEAR Serenity	History of statin intolerance to ≥ 2 statin LDL-C ≥ 130 mg/dL for primary prevention LDL-C ≥ 100 mg/dL for secondary prevention in ASCVD or for HeFH	Bempedoic acid 180 mg (*n* = 234) versus placebo (*n* = 111) Using low-dose statin (10%) Using other lipid-lowering agents (30%) Never used lipid-lowering agents (57%)	−21.4%
CLEAR Tranquility	History of statin intolerance with or without use of low low-dose statin LDL ≥ 100 mg/dL	Bempedoic acid 180 mg plus ezetimibe 10 mg (*n* = 181) versus placebo plus ezetimibe 10 mg (*n* = 88) Using low-dose statin (30%)	−28.5%

ASCVD: atherosclerotic cardiovascular disease, CLEAR: Cholesterol Lowering via ETC-1002, an ACL (ATP-citrate lyase)-Inhibiting Regimen, HeFH: heterozygous familial hypercholesterolemia, hs-CRP: high-sensitivity C-reactive protein, LDL-C: low-density lipoprotein cholesterol. ^#^ Maximally tolerated statin therapy: defined as the highest-intensity statin regimen that a patient was able to maintain, as determined by the investigator.

## Data Availability

Not applicable.
